# 1,2-Bis[1-(3-methyl­sulfanyl-1,2,4-triazin-5-yl)ethyl­idene]diazane

**DOI:** 10.1107/S1600536809025033

**Published:** 2009-07-04

**Authors:** Mariusz Mojzych, Zbigniew Karczmarzyk, Zofia Urbańczyk-Lipkowska, Przemysław Kalicki

**Affiliations:** a Department of Chemistry, University of Podlasie, ul. 3 Maja 54, 08-110 Siedlce, Poland; b Institute of Organic Chemistry, Polish Academy of Sciences, ul. Kasprzaka 44/52, 01-224 Warsaw 42, POB 58, Poland

## Abstract

The mol­ecule of the title compound, C_12_H_14_N_8_S_2_, has an N—N *gauche* conformation. The triazine rings are nearly coplanar with respect to the imide bonds [C—C—C—N torsion angles = −15.3 (3) and −15.8 (3)°] and they are twisted by 77.88 (7)°. The overall conformation of the mol­ecule is stabilized by intra­molecular C—H⋯N hydrogen bonding. The mol­ecular packing is influenced by π–π inter­actions of the triazine systems with a shortest centroid–centroid separation of 3.5242 (12) Å.

## Related literature

For the biological activity of hydrazones, see: Rollas *et al.* (2002 ▶); Terzioglu & Gürsoy (2003 ▶); Bedia *et al.* (2006 ▶). For the synthesis, see: Karczmarzyk *et al.* (2000 ▶); Rykowski *et al.* (2000 ▶); Mojzych & Rykowski (2003 ▶). For related structures, see: Lewis *et al.* (2000 ▶); Sauro & Workentin (2001 ▶); Tai *et al.* (2008 ▶).
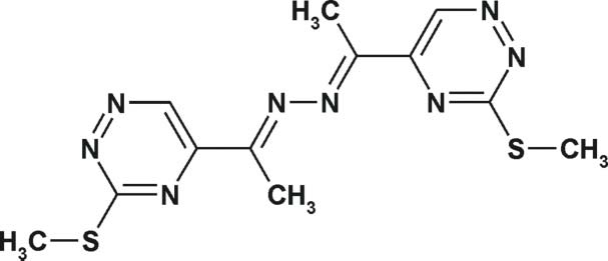


## Experimental

### 

#### Crystal data

C_12_H_14_N_8_S_2_
*M* *_r_* = 334.43
Monoclinic, 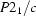

*a* = 14.4962 (4) Å
*b* = 7.0814 (2) Å
*c* = 15.6619 (5) Å
β = 107.465 (2)°
*V* = 1533.63 (8) Å^3^
*Z* = 4
Cu *K*α radiation
μ = 3.24 mm^−1^
*T* = 293 K
0.32 × 0.22 × 0.08 mm

#### Data collection

Bruker APEXII CCD diffractometer
Absorption correction: multi-scan (*SADABS*; Bruker, 2005 ▶) *T*_min_ = 0.474, *T*_max_ = 0.753
5750 measured reflections
2635 independent reflections
2200 reflections with *I* > 2σ(*I*)
*R*_int_ = 0.035

#### Refinement

*R*[*F* ^2^ > 2σ(*F* ^2^)] = 0.041
*wR*(*F* ^2^) = 0.117
*S* = 1.03
2635 reflections
199 parameters
H-atom parameters constrained
Δρ_max_ = 0.23 e Å^−3^
Δρ_min_ = −0.23 e Å^−3^

### 

Data collection: *APEX2* (Bruker, 2005 ▶); cell refinement: *SAINT* (Bruker, 2005 ▶); data reduction: *SAINT*; program(s) used to solve structure: *SHELXS97* (Sheldrick, 2008 ▶); program(s) used to refine structure: *SHELXL97* (Sheldrick, 2008 ▶); molecular graphics: *ORTEP-3 for Windows* (Farrugia, 1997 ▶); software used to prepare material for publication: *SHELXL97*, *WinGX* (Farrugia, 1999 ▶) and *PLATON* (Spek, 2009 ▶).

## Supplementary Material

Crystal structure: contains datablocks I, global. DOI: 10.1107/S1600536809025033/kp2223sup1.cif

Structure factors: contains datablocks I. DOI: 10.1107/S1600536809025033/kp2223Isup2.hkl

Additional supplementary materials:  crystallographic information; 3D view; checkCIF report

## Figures and Tables

**Table 1:** Hydrogen-bond geometry (Å, °)

*D*—H⋯*A*	*D*—H	H⋯*A*	*D*⋯*A*	*D*—H⋯*A*
C19—H193⋯N4	0.96	2.48	2.875 (3)	104
C20—H202⋯N8	0.96	2.46	2.816 (3)	101

**Fig. 1.**
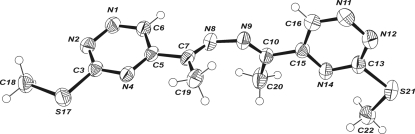
The molecular structure of (I) with atom labels and the 50% probability displacement ellipsoids for non-H atoms.

**Fig. 2.**
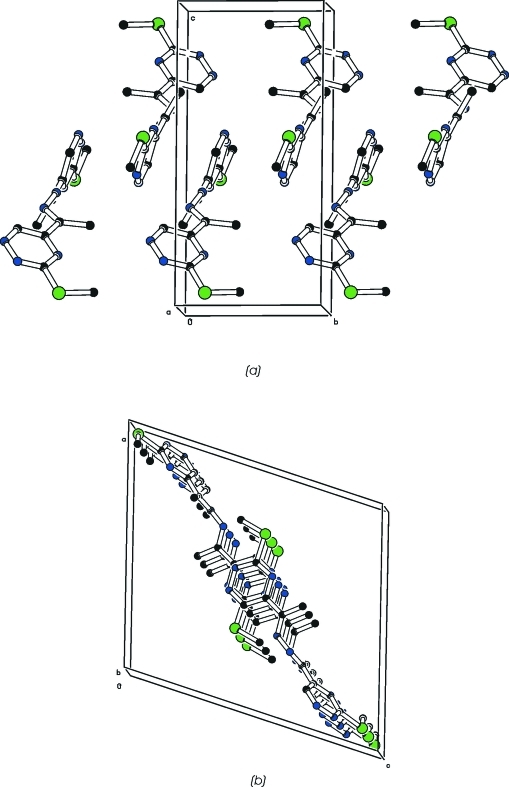
A view of part of the crystal structure of (I) along (a) [100] and (b) [010], showing the formation of a column of stacked triazine rings.

## supplementary crystallographic information

### Comment

Hydrazones have been found to possess antimicrobial, anticonvulsant, analgesic, antiinflammatory, antiplatelet, antitubercular, anticancer and antitumoral activities (Rollas *et al.*, 2002; Terzioglu & Gürsoy, 2003; Bedia *et al.*, 2006). Recently, we reported several aryl hydrazones of 5-acyl-1,2,4-triazine as important building blocks for the synthesis of *N*-1 substituted pyrazolo[4,3-*e*][1,2,4]triazines (Karczmarzyk *et al.*, 2000; Rykowski *et al.*, 2000). In continuation of work in this area we report herein the crystal structure of the title compound as intermediate for the construction of *N*-1 unsubstituted pyrazolo[4,3-*e*][1,2,4]triazines (Mojzych & Rykowski, 2003) and as a ligand for the synthesis of novel organometallic compounds with expected biological activity and other material applications (Sauro & Workentin, 2001).

In the title molecule, the C—N imide bonds of 1.283 (3) and 1.279 (3) Å and N—N hydrazine bond of 1.370 (2) Å are very similar to those reported for other related structures (Lewis *et al.*, 2000; Tai *et al.*, 2008). The molecule of (I) has an N—N *gauche* conformation with C7—N8—N9—C10 torsion angle of 114.8 (2)°. The triazine rings are nearly coplanar with the respective imide bonds [C6—C5—C7—N8 = -15.3 (3)° and C16—-C15—C10—N9 = -15.8 (3)°] and they are twisted by 77.88 (7)° with respect to each other. This conformation is stabilized by the intramolecular C19—H193···N4 and C20—H202···N8 hydrogen bonds (Table 1). The methylsulfanyl groups are in different conformations in respect to the mother triazine rings with the torsion angles N4—C3—S17—C18 and N14—C13—S21—C22 of 179.03 (15) and 7.0 (2)°, respectively. Significant π-π interactions are observed in the packing (Spek, 2009). The triazine N1···C6 rings form molecular stacks in the [010] direction with centroid-to-centroid separations of 3.5242 (12) (1 - *x*, -*y*, 1 - *z*) and 3.6473 (12) Å (1 - *x*, 1 - *y*, 1 - *z*) (Fig. 2) and slippages of 0.898 and 1.615 Å, respectively.

### Experimental

The synthesis of compound (I) and its analytical data (IR, ^1^H NMR) were described by Mojzych & Rykowski (2003). Crystals suitable for X-ray diffraction analysis were grown by slow evaporation of an ethanol solution.

### Refinement

All H atoms were located in a difference Fourier map and treated as riding on their C atoms [C—H distances of 0.93 Å (aromatic) and 0.96 Å (CH_3_)] with *U*_iso_(H) = 1.5*U*_eq_(C). The completeness of the reflection count on 0.94 level instead of expected 1.0 is caused by the poor diffraction due to unfavourable shape of the crystals of (I) (thin yellow plates).

### Crystal data

**Table:**

C_12_H_14_N_8_S_2_	*F*(000) = 696
*M**_r_* = 334.43	*D*_x_ = 1.448 Mg m^−^^3^
Monoclinic, *P*2_1_/*c*	Cu *K*α radiation, λ = 1.54178 Å
Hall symbol: -P 2ybc	Cell parameters from 1791 reflections
*a* = 14.4962 (4) Å	θ = 5.8–66.9°
*b* = 7.0814 (2) Å	µ = 3.24 mm^−^^1^
*c* = 15.6619 (5) Å	*T* = 293 K
β = 107.465 (2)°	Plate, yellow
*V* = 1533.63 (8) Å^3^	0.32 × 0.22 × 0.08 mm
*Z* = 4	

### Data collection

**Table:**

Bruker APEXII CCD diffractometer	2635 independent reflections
Radiation source: fine-focus sealed tube	2200 reflections with *I* > 2σ(*I*)
graphite	*R*_int_ = 0.035
φ and ω scans	θ_max_ = 67.7°, θ_min_ = 5.8°
Absorption correction: multi-scan (*SADABS*; Bruker, 2005)	*h* = −17→17
*T*_min_ = 0.474, *T*_max_ = 0.753	*k* = −8→2
5750 measured reflections	*l* = −18→17

### Refinement

**Table:**

Refinement on *F*^2^	Primary atom site location: structure-invariant direct methods
Least-squares matrix: full	Secondary atom site location: difference Fourier map
*R*[*F*^2^ > 2σ(*F*^2^)] = 0.041	Hydrogen site location: difference Fourier map
*wR*(*F*^2^) = 0.117	H-atom parameters constrained
*S* = 1.03	*w* = 1/[σ^2^(*F*_o_^2^) + (0.0575*P*)^2^ + 0.4463*P*] where *P* = (*F*_o_^2^ + 2*F*_c_^2^)/3
2635 reflections	(Δ/σ)_max_ < 0.001
199 parameters	Δρ_max_ = 0.23 e Å^−^^3^
0 restraints	Δρ_min_ = −0.22 e Å^−^^3^

### Special details

**Table:**

Geometry. All e.s.d.'s (except the e.s.d. in the dihedral angle between two l.s. planes) are estimated using the full covariance matrix. The cell e.s.d.'s are taken into account individually in the estimation of e.s.d.'s in distances, angles and torsion angles; correlations between e.s.d.'s in cell parameters are only used when they are defined by crystal symmetry. An approximate (isotropic) treatment of cell e.s.d.'s is used for estimating e.s.d.'s involving l.s. planes.
Refinement. Refinement of *F*^2^ against ALL reflections. The weighted *R*-factor *wR* and goodness of fit *S* are based on *F*^2^, conventional *R*-factors *R* are based on *F*, with *F* set to zero for negative *F*^2^. The threshold expression of *F*^2^ > σ(*F*^2^) is used only for calculating *R*-factors(gt) *etc*. and is not relevant to the choice of reflections for refinement. *R*-factors based on *F*^2^ are statistically about twice as large as those based on *F*, and *R*- factors based on ALL data will be even larger.

### Fractional atomic coordinates and isotropic or equivalent isotropic displacement parameters (Å^2^)

**Table:**

	*x*	*y*	*z*	*U*_iso_*/*U*_eq_	
S17	0.29719 (4)	0.25053 (8)	0.43807 (4)	0.0484 (2)	
S21	0.94963 (5)	0.20147 (9)	0.04349 (4)	0.0524 (2)	
N1	0.56957 (14)	0.2978 (3)	0.57615 (13)	0.0434 (4)	
N2	0.47200 (14)	0.3040 (3)	0.55353 (12)	0.0416 (4)	
N4	0.46056 (13)	0.1897 (2)	0.40671 (11)	0.0369 (4)	
N8	0.69264 (14)	0.1583 (3)	0.38213 (12)	0.0430 (4)	
N9	0.74256 (14)	0.0796 (3)	0.32936 (13)	0.0437 (5)	
N11	0.92948 (16)	−0.1765 (3)	0.21425 (15)	0.0530 (5)	
N12	0.95071 (15)	−0.0836 (3)	0.14810 (14)	0.0513 (5)	
N14	0.86038 (13)	0.1842 (2)	0.17013 (12)	0.0391 (4)	
C3	0.42339 (16)	0.2505 (3)	0.47130 (15)	0.0365 (5)	
C5	0.55527 (16)	0.1885 (3)	0.42894 (14)	0.0346 (5)	
C6	0.61047 (17)	0.2431 (3)	0.51602 (15)	0.0384 (5)	
H61	0.6776	0.2403	0.5312	0.058*	
C7	0.60199 (16)	0.1214 (3)	0.36205 (14)	0.0373 (5)	
C10	0.77872 (16)	0.1880 (3)	0.28245 (14)	0.0399 (5)	
C13	0.91659 (16)	0.0920 (3)	0.13019 (15)	0.0410 (5)	
C15	0.83999 (15)	0.0913 (3)	0.23536 (14)	0.0374 (5)	
C16	0.87630 (17)	−0.0913 (3)	0.25750 (16)	0.0476 (6)	
H161	0.8624	−0.1544	0.3042	0.071*	
C18	0.27045 (19)	0.3325 (3)	0.53668 (18)	0.0524 (6)	
H181	0.2921	0.2408	0.5836	0.079*	
H182	0.2019	0.3504	0.5235	0.079*	
H183	0.3030	0.4502	0.5557	0.079*	
C19	0.54418 (18)	0.0180 (3)	0.28090 (15)	0.0470 (6)	
H191	0.5800	−0.0897	0.2712	0.071*	
H192	0.5309	0.1002	0.2299	0.071*	
H193	0.4844	−0.0232	0.2892	0.071*	
C20	0.7638 (2)	0.3954 (3)	0.27192 (19)	0.0583 (7)	
H201	0.8222	0.4541	0.2681	0.087*	
H202	0.7476	0.4451	0.3226	0.087*	
H203	0.7122	0.4211	0.2183	0.087*	
C22	0.9029 (2)	0.4349 (4)	0.04713 (18)	0.0561 (6)	
H221	0.8344	0.4284	0.0377	0.084*	
H222	0.9156	0.5109	0.0011	0.084*	
H223	0.9337	0.4904	0.1045	0.084*	

### Atomic displacement parameters (Å^2^)

**Table:**

	*U*^11^	*U*^22^	*U*^33^	*U*^12^	*U*^13^	*U*^23^
S17	0.0406 (3)	0.0580 (4)	0.0483 (4)	0.0010 (3)	0.0158 (3)	−0.0040 (3)
S21	0.0571 (4)	0.0614 (4)	0.0505 (4)	−0.0013 (3)	0.0341 (3)	−0.0052 (3)
N1	0.0458 (11)	0.0510 (10)	0.0343 (10)	−0.0001 (9)	0.0135 (8)	−0.0036 (8)
N2	0.0461 (11)	0.0460 (10)	0.0351 (10)	0.0023 (8)	0.0157 (8)	−0.0016 (8)
N4	0.0431 (10)	0.0387 (9)	0.0315 (9)	0.0004 (8)	0.0151 (8)	0.0010 (7)
N8	0.0475 (11)	0.0490 (10)	0.0386 (10)	−0.0007 (9)	0.0224 (9)	−0.0009 (8)
N9	0.0470 (11)	0.0506 (10)	0.0401 (10)	0.0022 (9)	0.0232 (9)	−0.0007 (8)
N11	0.0533 (12)	0.0532 (11)	0.0548 (12)	0.0129 (10)	0.0196 (10)	0.0027 (10)
N12	0.0525 (12)	0.0544 (11)	0.0522 (12)	0.0094 (9)	0.0237 (10)	−0.0023 (9)
N14	0.0382 (10)	0.0453 (10)	0.0372 (10)	−0.0016 (8)	0.0165 (8)	−0.0044 (8)
C3	0.0428 (12)	0.0319 (10)	0.0381 (12)	0.0010 (9)	0.0172 (10)	0.0033 (8)
C5	0.0435 (12)	0.0299 (9)	0.0340 (11)	−0.0010 (9)	0.0169 (9)	0.0039 (8)
C6	0.0389 (12)	0.0422 (11)	0.0357 (11)	−0.0008 (9)	0.0139 (9)	−0.0008 (9)
C7	0.0493 (13)	0.0349 (10)	0.0320 (11)	0.0016 (9)	0.0188 (9)	0.0052 (8)
C10	0.0391 (12)	0.0493 (12)	0.0337 (11)	0.0012 (9)	0.0143 (9)	−0.0020 (9)
C13	0.0375 (12)	0.0494 (12)	0.0392 (12)	−0.0022 (10)	0.0161 (9)	−0.0091 (9)
C15	0.0343 (11)	0.0458 (11)	0.0323 (11)	−0.0018 (9)	0.0105 (9)	−0.0030 (9)
C16	0.0470 (14)	0.0528 (13)	0.0440 (13)	0.0081 (10)	0.0154 (11)	0.0057 (10)
C18	0.0534 (15)	0.0535 (13)	0.0598 (16)	0.0080 (11)	0.0313 (13)	0.0023 (12)
C19	0.0565 (15)	0.0510 (13)	0.0373 (12)	−0.0026 (11)	0.0198 (11)	−0.0064 (10)
C20	0.0782 (19)	0.0466 (13)	0.0662 (17)	0.0049 (13)	0.0459 (15)	−0.0011 (12)
C22	0.0527 (15)	0.0635 (15)	0.0593 (16)	0.0019 (12)	0.0280 (13)	0.0089 (12)

### Geometric parameters (Å, °)

**Table:**

S17—C3	1.745 (2)	C6—H61	0.9300
S17—C18	1.798 (2)	C7—C19	1.488 (3)
S21—C13	1.751 (2)	C10—C15	1.481 (3)
S21—C22	1.794 (3)	C10—C20	1.487 (3)
N1—C6	1.313 (3)	C15—C16	1.400 (3)
N1—N2	1.352 (3)	C16—H161	0.9300
N2—C3	1.324 (3)	C18—H181	0.9600
N4—C5	1.311 (3)	C18—H182	0.9600
N4—C3	1.352 (3)	C18—H183	0.9600
N8—C7	1.283 (3)	C19—H191	0.9600
N8—N9	1.370 (2)	C19—H192	0.9600
N9—C10	1.279 (3)	C19—H193	0.9600
N11—C16	1.315 (3)	C20—H201	0.9600
N11—N12	1.339 (3)	C20—H202	0.9600
N12—C13	1.337 (3)	C20—H203	0.9600
N14—C15	1.321 (3)	C22—H221	0.9600
N14—C13	1.337 (3)	C22—H222	0.9600
C5—C6	1.412 (3)	C22—H223	0.9600
C5—C7	1.486 (3)		
			
C3—S17—C18	102.76 (12)	N14—C15—C10	117.49 (19)
C13—S21—C22	100.93 (11)	C16—C15—C10	122.7 (2)
C6—N1—N2	118.93 (19)	N11—C16—C15	122.1 (2)
C3—N2—N1	117.08 (18)	N11—C16—H161	119.0
C5—N4—C3	115.11 (18)	C15—C16—H161	119.0
C7—N8—N9	117.19 (19)	S17—C18—H181	109.5
C10—N9—N8	118.97 (18)	S17—C18—H182	109.5
C16—N11—N12	118.7 (2)	H181—C18—H182	109.5
C13—N12—N11	117.58 (19)	S17—C18—H183	109.5
C15—N14—C13	115.32 (19)	H181—C18—H183	109.5
N2—C3—N4	127.1 (2)	H182—C18—H183	109.5
N2—C3—S17	119.62 (17)	C7—C19—H191	109.5
N4—C3—S17	113.23 (17)	C7—C19—H192	109.5
N4—C5—C6	119.94 (19)	H191—C19—H192	109.5
N4—C5—C7	118.52 (19)	C7—C19—H193	109.5
C6—C5—C7	121.5 (2)	H191—C19—H193	109.5
N1—C6—C5	121.8 (2)	H192—C19—H193	109.5
N1—C6—H61	119.1	C10—C20—H201	109.5
C5—C6—H61	119.1	C10—C20—H202	109.5
N8—C7—C19	125.6 (2)	H201—C20—H202	109.5
N8—C7—C5	114.34 (19)	C10—C20—H203	109.5
C19—C7—C5	120.0 (2)	H201—C20—H203	109.5
N9—C10—C15	114.78 (19)	H202—C20—H203	109.5
N9—C10—C20	125.9 (2)	S21—C22—H221	109.5
C15—C10—C20	119.33 (19)	S21—C22—H222	109.5
N12—C13—N14	126.5 (2)	H221—C22—H222	109.5
N12—C13—S21	113.86 (16)	S21—C22—H223	109.5
N14—C13—S21	119.58 (17)	H221—C22—H223	109.5
N14—C15—C16	119.80 (19)	H222—C22—H223	109.5
			
C6—N1—N2—C3	1.1 (3)	C6—C5—C7—C19	163.73 (19)
C7—N8—N9—C10	114.8 (2)	N8—N9—C10—C15	173.76 (19)
C16—N11—N12—C13	0.3 (3)	N8—N9—C10—C20	−6.5 (4)
N1—N2—C3—N4	0.1 (3)	N11—N12—C13—N14	−2.1 (4)
N1—N2—C3—S17	178.80 (15)	N11—N12—C13—S21	179.33 (17)
C5—N4—C3—N2	−1.6 (3)	C15—N14—C13—N12	2.1 (3)
C5—N4—C3—S17	179.68 (14)	C15—N14—C13—S21	−179.36 (16)
C18—S17—C3—N2	0.15 (19)	C22—S21—C13—N12	−174.30 (19)
C18—S17—C3—N4	179.03 (15)	C22—S21—C13—N14	7.0 (2)
C3—N4—C5—C6	1.8 (3)	C13—N14—C15—C16	−0.5 (3)
C3—N4—C5—C7	179.32 (16)	C13—N14—C15—C10	179.41 (19)
N2—N1—C6—C5	−0.8 (3)	N9—C10—C15—N14	164.3 (2)
N4—C5—C6—N1	−0.7 (3)	C20—C10—C15—N14	−15.4 (3)
C7—C5—C6—N1	−178.20 (18)	N9—C10—C15—C16	−15.8 (3)
N9—N8—C7—C19	−7.1 (3)	C20—C10—C15—C16	164.4 (2)
N9—N8—C7—C5	171.86 (17)	N12—N11—C16—C15	1.2 (4)
N4—C5—C7—N8	167.24 (18)	N14—C15—C16—N11	−1.1 (4)
C6—C5—C7—N8	−15.3 (3)	C10—C15—C16—N11	179.0 (2)
N4—C5—C7—C19	−13.8 (3)		

### Hydrogen-bond geometry (Å, °)

**Table:**

*D*—H···*A*	*D*—H	H···*A*	*D*···*A*	*D*—H···*A*
C19—H193···N4	0.96	2.48	2.875 (3)	104
C20—H202···N8	0.96	2.46	2.816 (3)	101
